# Genetic background and embryonic temperature affect DNA methylation and expression of *myogenin* and muscle development in Atlantic salmon (*Salmo salar*)

**DOI:** 10.1371/journal.pone.0179918

**Published:** 2017-06-29

**Authors:** Erik Burgerhout, Maren Mommens, Hanne Johnsen, Arnfinn Aunsmo, Nina Santi, Øivind Andersen

**Affiliations:** 1 Nofima AS, Tromsø, Norway; 2 AquaGen AS, Trondheim, Norway; 3 Nofima AS, Ås, Norway; 4 Department of Animal and Aquaculture Sciences, Norwegian University of Life Sciences, Ås, Norway; Universitat de Barcelona, SPAIN

## Abstract

The development of ectothermic embryos is strongly affected by incubation temperature, and thermal imprinting of body growth and muscle phenotype has been reported in various teleost fishes. The complex epigenetic regulation of muscle development in vertebrates involves DNA methylation of the *myogenin* promoter. Body growth is a heritable and highly variable trait among fish populations that allows for local adaptations, but also for selective breeding. Here we studied the epigenetic effects of embryonic temperature and genetic background on body growth, muscle cellularity and *myogenin* expression in farmed Atlantic salmon (*Salmo salar*). Eggs from salmon families with either high or low estimated breeding values for body growth, referred to as Fast and Slow genotypes, were incubated at 8°C or 4°C until the embryonic ‘eyed-stage’ followed by rearing at the production temperature of 8°C. Rearing temperature strongly affected the growth rates, and the 8°C fish were about twice as heavy as the 4°C fish in the order Fast8>Slow8>Fast4>Slow4 prior to seawater transfer. Fast8 was the largest fish also at harvest despite strong growth compensation in the low temperature groups. Larval *myogenin* expression was approximately 4–6 fold higher in the Fast8 group than in the other groups and was associated with relative low DNA methylation levels, but was positively correlated with the expression levels of the DNA methyltransferase genes *dnmt1*, *dnmt3a* and *dnmt3b*. Juvenile Fast8 fish displayed thicker white muscle fibres than Fast4 fish, while Slow 8 and Slow 4 showed no difference in muscle cellularity. The impact of genetic background on the thermal imprinting of body growth and muscle development in Atlantic salmon suggests that epigenetic variation might play a significant role in the local adaptation to fluctuating temperatures over short evolutionary time.

## Introduction

Ambient temperature controls and limits virtually all biochemical and physiological processes and behavioural activities in poikilothermic organisms. While heat stress during organogenesis may be teratogenic also to poikilothermic embryos, they show phenotypic plasticity to moderate changes in incubation temperature that strongly affect embryonic development and may have persistent effects on various phenotypic traits. Ducklings reared at relative low temperature showed slow growth and reduced thermoregulatory capacity after hatching [1], while embryonic temperature influenced growth rate and temperature choice in juvenile snapping turtles (*Chelydra serpentine*) [2] and swimming performance in wood frog tadpoles (*Rana sylvatica*) [3]. In zebrafish (*Danio rerio*), early temperature strongly affected metabolic enzymes in the skeletal muscle, swimming performance and thermal acclimation capacity of the adult fish [4,5]. In most teleosts, both hypertrophic and hyperplastic muscle growth continue after hatching [6–8], and the muscle phenotype at later stages has been shown to be programmed by embryonic temperature in various species [9–14]. The lasting effects of embryonic temperature on muscle have been demonstrated to involve temperature-dependent changes in the number of muscle precursor cells, which are responsible for postembryonic growth in teleosts [11,15,16].

Skeletal muscle development in vertebrates is regulated by the concerted action of the four myogenic regulatory factors (MRFs) MyoD, myf5, myogenin and MRF4. Mice knockout studies revealed that MyoD and myf5 are required to specify myoblasts, while myogenin and MRF4 act later to mediate differentiation of myoblasts into and fusion of myotubes [17–21]. Thermal effects on the embryonic expression of *MyoD* and *myogenin* have been reported in various teleosts, including Atlantic salmon and rainbow trout (*Oncorhynchus mykiss*) [22–24]. The upregulated *myogenin* expression and increased muscle development at elevated temperatures in Senegalese sole (*Solea senegalensis*) were associated with decreased methylation of the *myogenin* promoter and decreased expression of the DNA methyltransferases *dnmt1* and *dnmt3b* [25]. Expression of *myogenin* was consistently downregulated at low temperatures in turkey embryos and in mouse skeletal muscle cells *in vitro* [26,27]. Further, demethylation of the mouse *myogenin* promoter activated the gene expression in somites during skeletal muscle differentiation [28,29], and inhibition of DNA methylation in mesenchymal and dental pulp stem cells up-regulated *myogenin* expression and induced myogenesis [30,31]. The complex epigenetic network regulating muscle development also involves histone modifications and myogenic microRNAs controlling the expression of key myogenic factors [32–36].

Studies linking genetic variation and epigenetic regulation have mainly been conducted in plants, yeast and *Drosophila*, but the inheritance of epigenetic alleles have also been reported in mice displaying different coat colour phenotypes, in niche-adapted Darwin finches, and in association with various human diseases [37–40]. Intriguingly, differences in adaptive phenotypic responses, including body size, to climate changes enabled by epigenetics were recently reported in two populations of winter skate (*Leucoraja ocellata*) [41]. Migratory salmonids have a tendency to spawn in their homing rivers differing in seasonal and regional thermal conditions, and evidence of local genetic adaptation in muscle cellularity and body growth have been reported in wild populations [11,42,43]. Substantial heritability in body growth exists in Atlantic salmon, and significant enhancements in growth rate and hence reduction in production time have been achieved in farmed Atlantic salmon through 11 generations of selective breeding since 1972 [44]. Here we examine the impact of genetic background and embryonic temperature on the epigenetic regulation of body growth and white muscle phenotype in farmed Atlantic salmon.

## Material and methods

### Experimental set up

**Fig 1:**
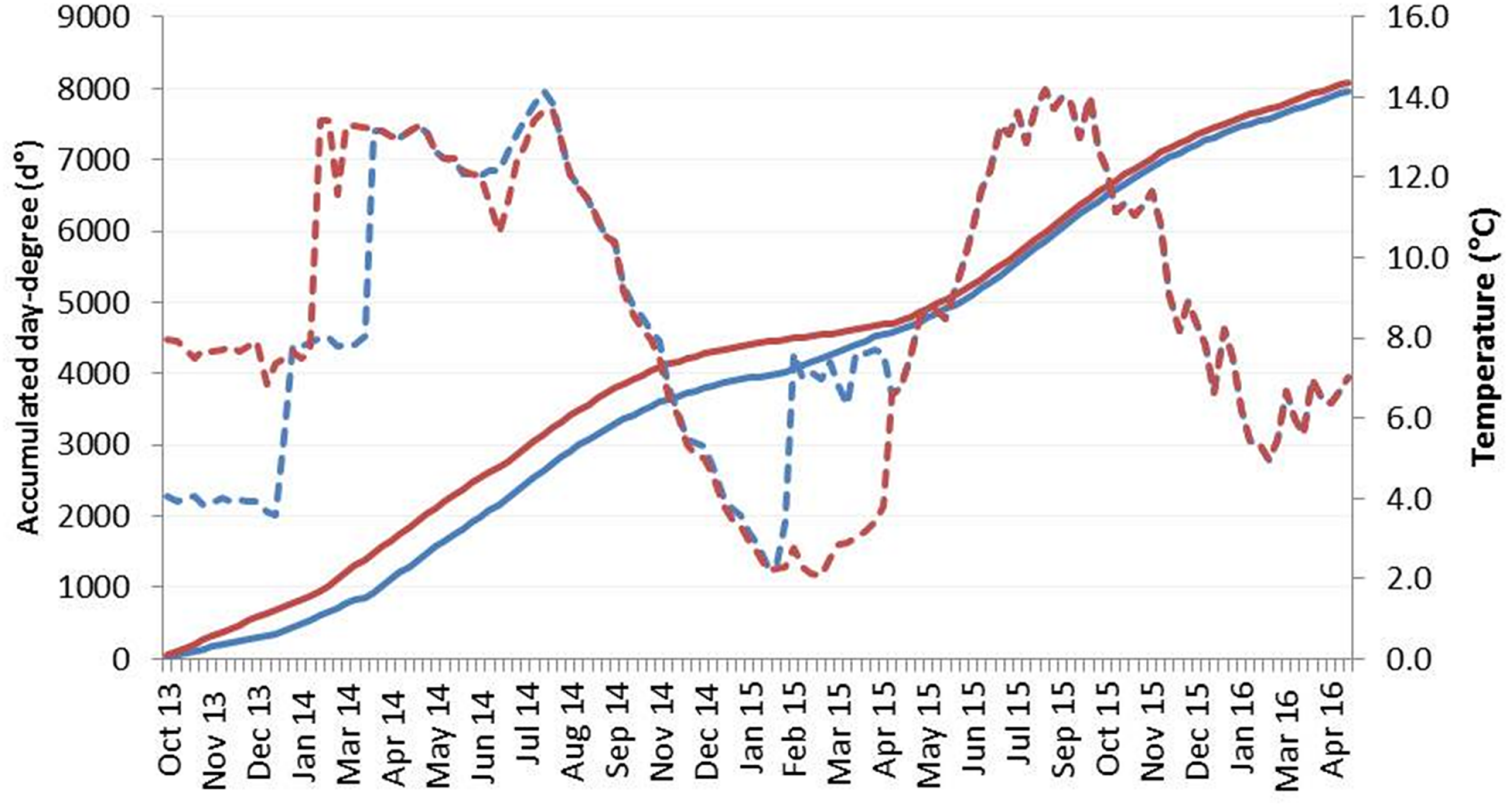
Temperature and day-degrees profiles. Temperature (°C, striped line) and accumulated day-degrees (d°, solid line) profiles between fertilization and harvest for the low (4°C, blue) and high (8°C, red) embryonic temperature groups. All fish were kept at the same temperature from the eyed-stage, except for the elevated temperature in the Fast4 and Slow4 groups prior to sea transfer. Details are given in Material and Methods.

Families with fast and slow on-growth within the natural variation were selected from AquaGen’s breeding nucleus year class 2011 (11^th^ generation with a heritability h^2^ of 0.3) by estimating breeding values (EBV) based on weight registrations at tagging, smolt weight, and harvest weight of the year class. Eight families with a mean EBV of 1.6 standard deviations (SD) higher than average and eight families with 1.6 SD lower than average were chosen and referred to as Fast and Slow genotypes, respectively. The families were created from eight different dams and fourteen different sires. The eight dams used to produce fast on-growth families originated from five families and the sires from seven families, while the eight dams used to produce slow on-growth families originated from three families and the sires from seven families. From October 2013, 600 fertilized eggs from each family were incubated at 4°C (3.9 ± 0.2°C) or 8°C (7.6 ± 0.5°C) until the eyed stage (360 day degrees, d° = number of days x temperature) that resulted in four experimental groups referred to as Fast4, Fast8, Slow4 and Slow8. Low-quality embryos were removed after physical shocking at 316 d° according to AquaGen’s standard protocol, and one of the Slow families was excluded due to high mortality at this stage. Dead and pin-eyed embryos were removed at the eyed stage, and the incubation temperature of the Fast4 and Slow4 groups was gradually increased to the standard production temperature of 8°C over three days. All groups were kept at this temperature until start feeding at which stage the temperature was further increased to 12.2 ± 1.1°C. Juveniles at the parr stage were RFID (radio frequency ID) tagged for individual identification at body weight of ~25 g (Fast8, Slow8) or ~8 g (Fast4, Slow4). From August 2014, a maximum of 200 individuals from each experimental group were kept for further on-growth at ambient temperatures varying from 2°C to 14°C in a flow-through system until sea transfer in April 2015 (Fig 1). For all groups to be transferred into seawater at similar total d° (calculated from fertilization to sea transfer), we increased the temperature in the Fast4 and Slow4 groups from ~2 to ~7°C for a period of 9 weeks prior to sea transfer (Fig 1, S1 Table). All groups were kept together in one sea cage (about 3000–4000 m3) until harvest in April 2016. Fish were fed by appetite with EWOS feed from start feeding until harvest.

### Sampling

Body weight was measured in sub-samples at the start feeding, parr, and pre-smolt stages, while individual weight was recorded in smolts before sea transfer and in adults at harvest. All measurements were performed in fish anesthetized using metacain (Finquel vet., Scanvacc) in accordance to the supplier’s instructions. Prior to any sampling, fish were euthanized using an overdose of metacain. An overview of sampling dates and d° for each treatment group is provided in S1 Table. Muscle samples directly rostral of the dorsal fin were collected from start feeding larvae and parr of ~6g body weight, and fixated in RNA-later (Ambion) and in 96% ethanol. Samples in RNA-later were kept overnight at 4°C followed by storage at -20°C until further analysis, while samples in ethanol were stored at -20°C. For histology, a transversal section directly rostral of the dorsal fin was obtained from parr, fixed in 4% paraformaldehyde solution (PFA, Electron Microscopy Sciences), and were kept overnight at 4°C. Thereafter, samples were washed in phosphate buffered saline (PBS, Sigma-Aldrich) for 10 min, dehydrated in 50% ethanol (30 min) and 70% ethanol (2 x 1 hr), and stored at -20°C until further analysis.

Thermal growth coefficient (TGC; [45]) in freshwater and seawater was calculated by:

$$\text{TGC }=\;\left({\text{BW}}_{1}{\;}^{1/3}–{\text{BW}}_{2}{\;}^{1/3}\right)\text{ x }1000\text{ x }\Sigma {\text{T }}^{-1}$$

In the freshwater phase, TGC was calculated on mean weight (g) per treatment group from start feeding (BW2) to sea transfer (BW1), and d° using the temperature measured in tanks. In seawater phase, individual body weight (g) at sea transfer (BW2) and at harvest (BW1) was used, and d° using the temperature measured at 5m depth.

Standardized harvest weight (SHW) was used to standardize harvest weight independent of different smolt start weight at sea transfer [46]. SHW has been developed to standardize field trial weight data acquired in commercial Atlantic salmon farming where differences in production regimes can lead to uneven growth differences [47]. The more commonly used specific growth rate (SGR) is usually biased towards small fish when used to compare fish of uneven size. We used a smolt weight of 200 g, which was the lowest weight among the treated groups at transfer, and TGC combined with the sum of d° between sea transfer and harvest:

$$\text{SHW }=\;(200{\text{ g}}^{1/3}+\;{\left(\text{TGC}/\left(1000\;\times \;3353.5\right)\right)}^{3}$$

Difference in SHW (ΔSHW) for 4°C groups was calculated by:

ΔSHW = SHW(4°C) − SHW (8°C).

### DNA methylation analysis

Four families from each of the four treatment groups (n = 6 per family) were chosen for DNA methylation analysis. Total genomic DNA was isolated and purified from muscle tissue at the start feeding and parr stages using DNeasy Blood & Tissue Kit (Qiagen) according to the manufacturer’s protocol and DNA ethanol precipitation method, respectively, due to the differences in sample size. DNA quantity and quality were measured using a 1000-ND Nanodrop spectrophotometer (Nanodrop Technologies). DNA was bisulfite converted using the Epitect Fast Bisulfite Conversion Kit (Qiagen) according to the manufacturer’s protocol.

Six specific pyrosequencing assays (PyroMark Custom Assay, Qiagen) covering fourteen putative CpG sites identified in the Atlantic salmon *myogenin* promoter (NC_027321.1, S2 Fig) were designed using PyroMark Assay Design 2.0 (Qiagen) (S2 Table). Assay specific PCRs were performed using the PyroMark PCR Kit (Qiagen) according to manufacturer’s protocol. Singular PCR products were verified on a 1% agarose gel. Five of the fourteen CpG sites could be analyzed using pyrosequencing, as only two of the six primer sets (S2 Table) provided a singular PCR product. The PyroMark Q24 (Qiagen) was used in combination with the PyroMark 24 Advanced CpG Reagents (Qiagen) and Streptavidin Sepharose High Performance beads (GE Healthcare) to analyze the CpG methylation assays by pyrosequencing technology following the manufacturer’s PyroMark Q24 Advanced protocol using 25uL sequencing primer solution.

### Gene expression analysis

Four families from each of the four treatment groups (n = 6–10 per family) were chosen for gene expression analysis in fast muscle tissue at the start feeding and parr stages. Total RNA was extracted and purified using the MagMax-96 Total RNA Isolation Kit (Applied Biosystems) with the MagMax-96 Magnetic Particle Processor (Applied Biosystems) and AllPrep DNA/RNA/miRNA Universal kit (Qiagen), respectively, following manufacturer’s instructions. Both methods were used due to differences in samples size. RNA extracted using the MagMax-96 kit was followed by a clean-up using the Turbo DNase Treatment Kit (Applied Biosciences). RNA quantity and quality were measured using a 1000-ND Nanodrop spectrophotometer. cDNA was synthesized using the Vilo Superscript Kit (Invitrogen) following the manufacturer’s instructions using 200ng of total RNA. The relative expression levels of *myogenin*, the DNA methyltransferase genes *dnmt1* (XM_014193376), *dnmt3a* (XM_014136242.1), *dnmt3b* (XM_014146676.1) and the reference gene *elongation factor 1α* (*ef1α*, [48]) were determined using quantitative real-time PCR (qPCR), and primers were designed using Primer3 program (Applied Biosystems) (S1 Table). A two-fold standard dilution of muscle cDNA was set up for each primer set in order to determine the amplification efficiency. The qPCR was run in duplicates using the 7900HT Fast Real-Time PCR system (Applied Biosystems) with a total volume of 20μL containing 10μL Power SYBR Green PCR Master Mix (Applied Biosystems), 0.6μL 10μM forward and reverse primers, 8μL diluted cDNA. A cycling profile of 10 min at 95°C, followed by 40 cycles of 95°C for 15s, 60°C for 60s. Absence of genomic DNA was verified by running randomly chosen RNA samples. In order to rule out non-specific contamination a no template control was included and a melting curve analysis was performed to verify the measurement of a single specific product. SDS 2.3 software (Applied Biosystems) was used to collect all data that was thereafter analyzed using RQ manager 1.2 (Applied Biosystems). Relative gene expression was calculated based on the determined Ct values [49]. The treatment group with the lowest value was used as a calibrator and set to one (1).

### Muscle cellularity

Samples were dehydrated in ethanol series (2 times 96% for 1 hr and 2 times 100% for 1 hr), followed by clearing in Histoclear (National Diagnostics) for 1 hr, Histoclear/paraffin (1:2; Histowax, Histolab Products AB) and embedding in paraffin using a Citadel 2000 Tissue processor (Thermo Scientific). Sections of 7 μm were cut using a microtome (Leica RM2255), and were mounted on microscope slides. After rehydration, the samples were stained with haematoxylin-eosin (Shandon Instant Haematoxylin and Shandon Instant Eosin (alcoholic), Thermo Scientific)). Maximum length and width of ~200–400 cells (n = 10–12 per group) were measured in an area of 1 mm^2^ using ImageJ (https://imagej.nih.gov/ij). The fast muscle cell diameter (μm) was estimated indirectly by regarding the cell as a circle [25] using the following formula: diameter = (maximum length * maximum width)^1/2^. The relative distribution was expressed as: (number of cells at a certain diameter / the total number cells) * 100.

### Statistics

Data was tested for normal distribution using one-sample Kolmogorov-Smirnov statistic test. A general linear regression (GLM) was conducted to compare main effects of type of on-growth (fast, slow) and incubation temperature (4°C, 8°C) and the interaction between genotype and incubation temperature on body weight measured at the five developmental stages, and for TGC and SHW. Significant differences between experimental groups were analyzed by least square means (LS-means). As data for the expression and methylation levels was not normally distributed (K-S test p<0.05) a non-parametric Kruskall-Wallis test was performed following by Mann-WhitneyU test as posthoc test. Correlation analysis of the methylation sites and the expression data was performed using a non-parametric Spearman test. A student t-test was used to compare the average muscle fibre size between the groups. A two-sample Kolmogorov-Smirnov test was used to analyze differences in muscle fibre distributions. Data are presented as mean ± standard error and considered significantly different when p<0.05.

## Results

### Growth

Embryonic incubation at the low temperature of 4°C strongly delayed the developmental progress, but the 4°C and 8°C incubation groups reached the eyed stage and start feeding stage at quite similar number of d° (Fig 1, S3 Table). At start feeding the Fast8 and Slow8 fish were significantly heavier (0.19 g) than Fast4 and Slow4 (0.17 g), but the 4°C groups were heavier than the 8°C groups at the parr stage that was reached after 2085 d° and 1985 d°, respectively (Table 1). The body weight prior to seawater transfer was strongly influenced by both genotype and embryonic temperature, and the 8°C smolts were about twice as heavy as the 4°C smolts in the order Fast8>Fast4>Slow8>Slow4 after 4597 d° and 4313 d°, respectively. TGC in the freshwater phase was significantly higher in the high temperature groups (Fast8: 1.6, Slow8: 1.5) than in the low temperature groups (Fast4: 1.2, Slow4: 1.2), but the opposite was found in seawater (Fast8: 3.0 *vs* Fast4: 3.3, Slow8: 2.6 *vs* Slow4: 3.0) (Table 1). The catch-up growth of the 4°C fish in seawater reduced the weight difference between the 8°C and 4°C groups, and at harvest the Fast8 fish was 8% heavier than the Fast4 fish, while Slow8 was only 4% heavier than Slow4. SHW was significantly higher in both 4°C groups compared to 8°C groups that resulted in an estimated difference ΔSHW of 858 ± 45 g and 1019 ± 77 g for Fast4 and Slow4, respectively (Table 1).

**Table 1:** Biometrics.

	Fast 8		Fast 4		Slow 8		Slow 4	
**Start-feed**	0.19±0.005^a^	(96)	0.17±0.004^b^	(96)	0.19±0.005^a^	(82)	0.17±0.005^b^	(84)
**Parr**	5.9±0.2^bc^	(79)	7.0±0.2^a^	(80)	5.5±0.2^c^	(70)	6.5±0.2^ab^	(70)
**Pre-smolt**	247.6±5.7^b^	(81)	233.9±5.8^b^	(80)	239.4±6.9^b^	(56)	195.3±6.9^a^	(56)
**Smolt**	446±2^d^	(1275)	227±2^b^	(1339)	417±3^c^	(613)	200±2^a^	(950)
**TGC FW**	1.6±0.02^b^	(8)	1.2±0.02^a^	(8)	1.5±0.02^c^	(7)	1.2±0.02^a^	(7)
**Harvest**	5458±36^c^	(905)	5049±33^b^	(1061)	4234±55^a^	(382)	4084±38^a^	(800)
**TGC SW**	3.0±0.0^b^	(899)	3.3±0.0^a^	(1057)	2.6±0.0^c^	(382)	3.0±0.0^b^	(800)
**SHW**	4032±30^b^	(899)	4875±28^a^	(1057)	3105±46^c^	(380)	4123±32^b^	(798)
**ΔSHW**			858±45^a^	(8)			1019±77^a^	(7)

Body weight (g) and growth estimates from start-feeding until harvest of Fast and Slow genotypes reared at 4°C or 8°C until the eyed stage given as mean ± SE (number of individuals).

Weight estimates are based on sub-samples of each family from start-feeding to pre-smolt, and on individual weight registrations from smolt until harvest.

Thermal growth coefficient (TGC) and standardized harvest weight (SHW) are estimated from individual weight registrations, while ΔSHW is estimated from mean SHW in each family.

Different letters indicate significant differences between groups (p<0.05).

### DNA methylation of myogenin promoter

**Fig 2:**
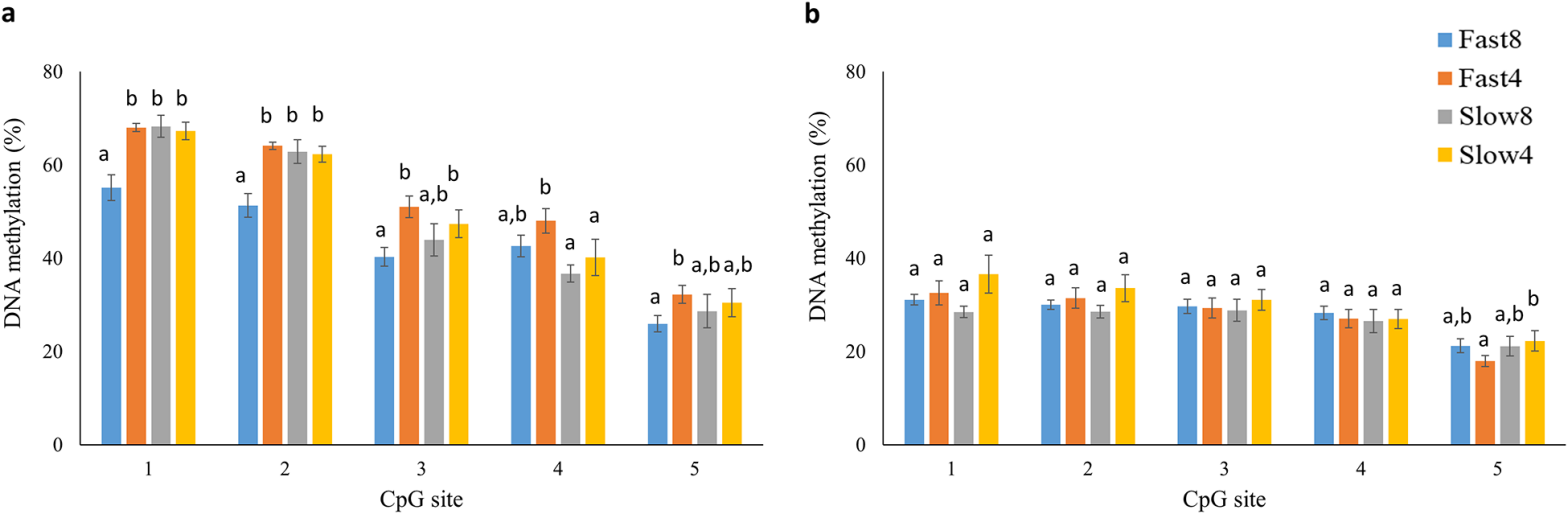
DNA methylation of *myogenin* promoter. DNA methylation (%) of five CpG sites in the promoter region of the *myogenin* gene measured in start feeding larvae (a) and in parr (b) reared at 4°C or 8°C until the eyed stage. Fast and Slow denote the genetic background of the fish. Numbers 1–5 correspond to the CpG sites at position -610, -598, -258, -255 and -234, respectively. Different letters indicate significant differences between groups (p<0.05).

DNA methylation of the *myogenin* promoter was assessed in muscle tissue of start feeding larvae and parr juveniles by pyrosequencing five CpG sites upstream of the translation start site. At start feeding the Fast8 group showed significantly lower DNA methylation level at positions -610 and -598 than in the other groups, and also at position -258 when compared with Fast4 and Slow4 (Fig 2a). In addition, Fast8 showed significantly lower methylation level than Fast4 at position -234, while the methylation at position -255 was lower in Slow4 and Slow8 than in Fast4. The DNA methylation of the five sites was significantly lower in parr than in start feeding larvae (Fig 2b). The four treatment groups showed less variation at the parr stage, and only position -234 showed a significantly lower level in the Fast4 compared with the Slow4 group (p<0.05).

### Gene expression patterns

**Fig 3:**
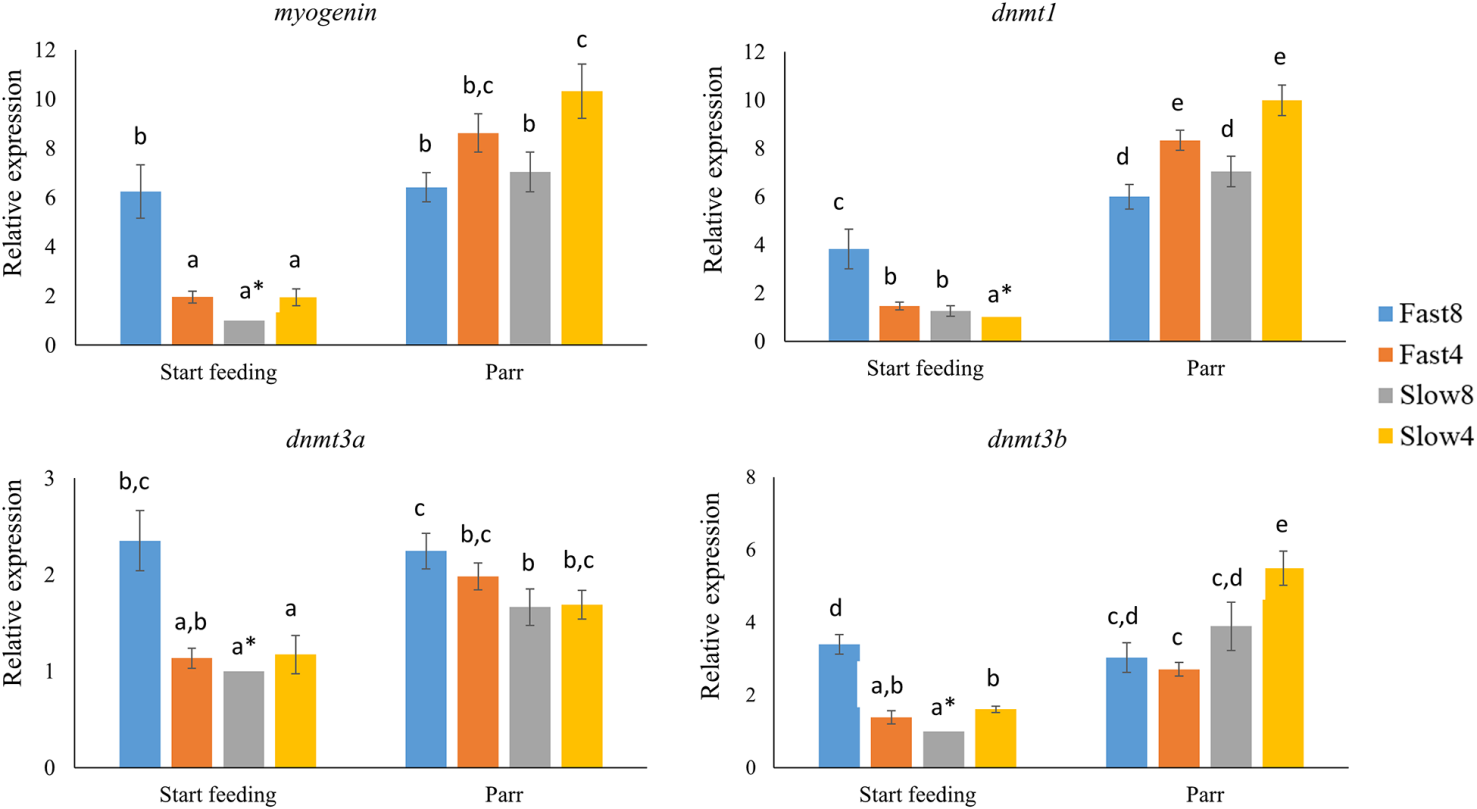
Gene expression of myogenin and DNA methyltransferases. Relative expression of *myogenin* and the DNA methyltransferases *dnmt1*, *dnmt3a* and *dnmt3b* in start feeding larvae and in parr from families with Fast and Slow genotypes after incubation at 4°C or 8°C until the eyed stage. Asterisks represent the calibrator group with the lowest expression value set to 1 for the gene expression analysis. Different letters indicate significant differences between groups and stages (p<0.05).

The relative expression levels of *myogenin* and the DNA methyltransferases *dnmt1*, *dnmt3a* and *dnmt3b* were determined in muscle tissue of start feeding larvae and parr using qPCR. At the larval stage, the mRNA levels of *myogenin* was approximately 4- to 6-fold higher in the Fast 8 group than in the other groups (p<0.05), in which no significant differences were found (Fig 3a). Similarly, Fast8 larvae expressed the *dnmt1*, *dnmt3a* and *dnmt3b* genes at significant higher levels than the other groups, except for the non-significant difference in *dnmt3a* mRNA levels between Fast8 and Fast4 (Fig 3b–3d). The four genes examined were expressed at higher levels at the parr stage than in the start-feeding larvae, except for the Fast8 parr displaying elevated levels of only *dnmt1* (p<0.05; Fig 3a–3d). The Slow4 parr expressed *myogenin*, *dnmt1* and *dnmt3b* at significantly higher levels than Fast 8 and Slow 8, while the *dnmt3a* expression was significantly higher in Fast8 compared Slow8.

Independent of stage, growth rate and rearing temperature, positive correlations were found between the expression of *myogenin* and *dnmt1* (ρ = 0.839; p<0.001), *myogenin* and *dnmt3a* (ρ = 0.762; p<0.001), *myogenin* and *dnmt3b* (ρ = 0.770; p<0.001) (S4 Table). Negative correlations were found between the methylation levels of the five studied sites and the expression of *myogenin*, *dnmt1*, *dnmt3a* and *dnmt3b*.

### Muscle cellularity

**Fig 4:**
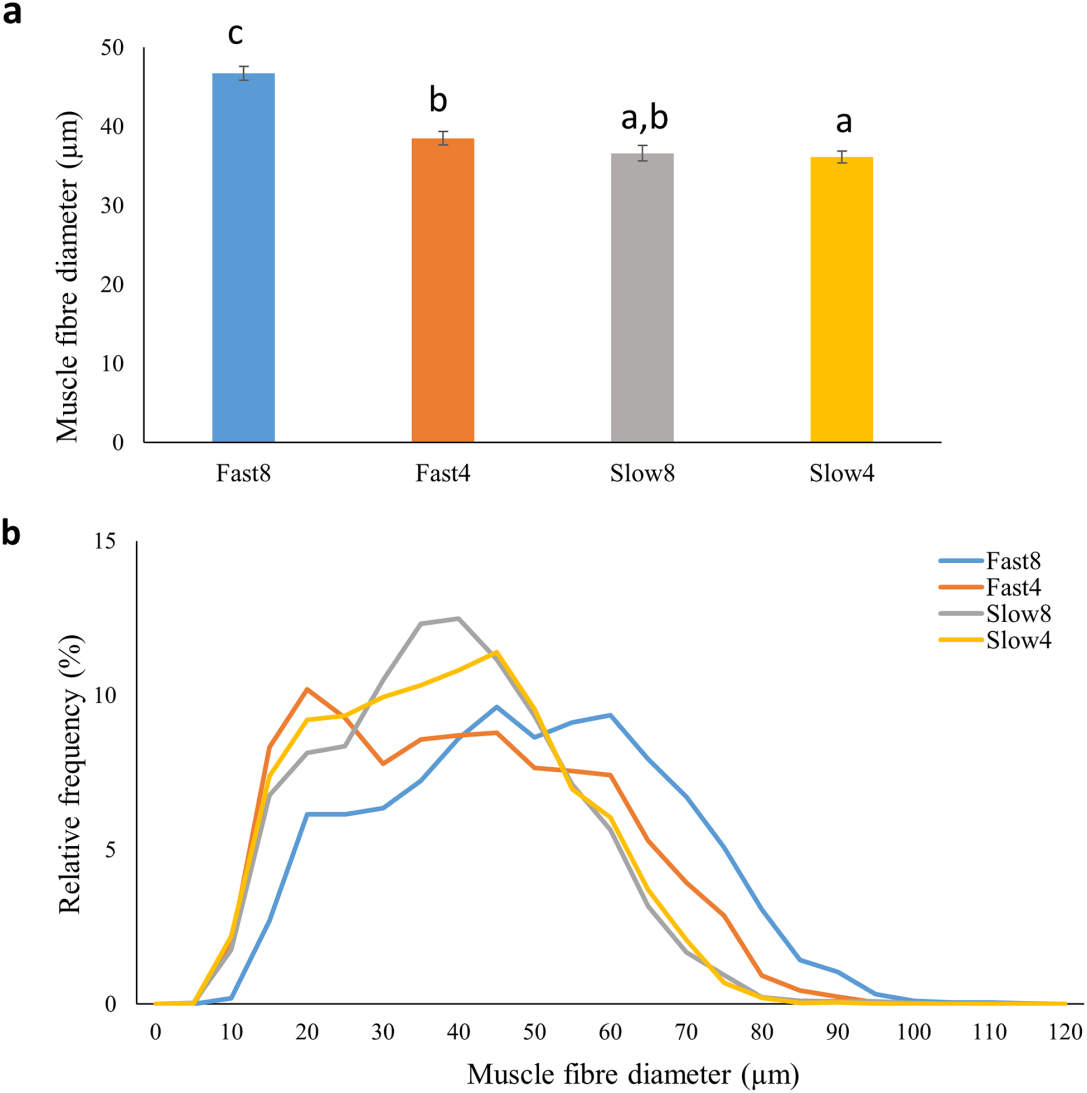
White muscle cellularity in Atlantic salmon parr. a) Average fibre diameter (μm), and b) size distribution (%) of fibre diameter in parr from families with Fast and Slow genotypes after incubation at 4°C or 8°C until the eyed-stage. Different letters present significant differences (p<0.05) between groups.

The average diameter of white muscle fibres was significantly larger in Fast8 compared to the other three groups at the parr stage, and also larger in Fast4 compared with Slow4 (p<0.05) (Fig 4a). The distribution of muscle fibre diameters (Fig 4b) showed that Fast8 possessed relatively more muscle fibres with a larger diameter than the other treatment groups (p<0.05; right-hand tail). Fast4 showed a distribution more towards the right-hand tail only compared to Slow4 (p<0.05).

## Discussion

This study demonstrated substantial effects of genetic background and embryonic temperature on muscle phenotype and body growth in farmed Atlantic salmon. While the temperature effects on body growth differed between the freshwater and seawater phases, fish from families of the Fast genotype were significantly heavier from the smolt stage until harvest than those with the Slow genotype. Independent of genotype, the high temperature fish were a two-fold heavier prior to seawater transfer than the low temperature fish, while the latter Fast4 and Slow4 fish grew significantly faster than Fast8 and Slow8 fish under the same conditions in seawater. The potential of compensatory growth experienced by the 4°C group was shown by using SHW as a model to standardize weight differences at sea transfer. The compensatory growth observed was probably resulting from different embryonic temperatures, although we cannot exclude possible effects of the temperature adjustments before seawater transfer and the timing of smoltification. Consistently, Atlantic salmon incubated at the low embryonic temperatures of 2°C or 5°C showed significant growth compensation after seawater transfer when compared to high temperature fish (8°C or 10°C) [14]. In comparison, salmon raised at embryonic temperature of 10°C prior to a decrease to 5°C grew faster than fish raised at 5°C during early stages, but the 5°C fish displayed higher foraging activity and a more sustained period of muscle growth [13]. Additionally, embryonic incubation at low temperature promoted hyperplasia in the white muscle of salmon larvae and juveniles [13,14,50], in agreement with the smaller fibre diameters in the Fast4 and Slow4 fish compared to Fast8 in the presents study. Intriguingly, Slow 8 and Slow4 showed no difference in muscle fibre thickness, suggesting that only the Fast genotype responded to elevated rearing temperature with hypertrophic growth. While large fibre size in fish skeletal muscle seems to be metabolically advantageous by reducing the cost of maintaining the membrane potential [51,52], a fast-growing strain of rainbow trout exhibited significantly smaller fibre diameter than a slow-growing strain from hatching to 24 cm body length that allowed for prolonged and greater muscle growth in the adult fish [53]. Consistently, pearlfish incubated at high embryonic temperature of 16°C showed reduced cell proliferation, but increased differentiation that gave rise to larger hatchlings, while their limited reserves of muscle precursor cells finally led to smaller adults than those incubated at 13 or 8.5°C [16].

Thermal imprinting of muscle cellularity involves modification of the proliferation and differentiation of the muscle precursor cells being regulated by conserved myogenic transcription factors [11,15,16]. Myogenin plays a crucial role in the differentiation of muscle precursor cells, and the ontogenic expression of *myogenin* in rainbow trout peaked in swim-up fry [54]. Correspondingly, the Fast8 fish displayed high *myogenin* expression at start-feeding, while the gene expression seemed to be delayed in the other treatment groups. Accordingly, low embryonic temperature delayed and prolonged expression of *MyoD*, *myogenin* and *MyHC* in rainbow trout that resulted in the recruitment of considerably more fibers compared to high temperature fish [22]. The elevated *myogenin* expression in the Fast8 fish coincided with the less methylated promoter region when compared to the other groups, in agreement with the inverse correlation between the *myogenin* expression and methylation levels in Senegalese sole at metamorphosis [25]. However, the expression of salmon *myogenin* was positively correlated with the gene expression of the DNA methyltransferases, in contrast to the negative correlation between the expression of *myogenin* and the levels of *dnmt1* and *dnmt3b* in Senegalese sole [25]. The function of Dnmt1 is maintenance of DNA methylation levels after cell division, while Dnmt3a and 3b are directly involved in *de novo* methylation [55,56]. The conflicting results could be explained by the simultaneous processes of methylation of a variety of genes, and thereby the effects on individual genes may become less clear. The epigenetic regulation of myogenesis is further complicated by the involvement of histone modifications and microRNAs [34,57], and histone methylation of three *pax7* genes was suggested to regulate *myogenin* expression in rainbow trout [35].

The thermal plasticity of muscle development and somatic growth in fish is thought to play a crucial role in the local adaptation to prevailing water temperature conditions, and evidence for genetic differences between populations has been provided in several species [11,12,58,59]. Eggs from two salmon populations spawning in a lowland or highland tributary of the Scottish River Dee System responded differently to the two temperature regimes when incubated together [11], while strong temperature and family effects on muscle cellularity were demonstrated in farmed families of Atlantic salmon originating from the Scottish River Shin [58]. The genetic gain for growth rate in Atlantic salmon has been estimated at 10–15% per generation [44], but no single SNP with a significant effect on growth has been identified across year-classes ([60], Moen et al. unpubl). The polygenic nature of this trait, together with the complex epigenetic mechanisms regulating skeletal muscle growth, makes it difficult to identify the mechanisms underlying the epigenetic variation found in Atlantic salmon.

## Conclusions

This study demonstrates strong interactions between thermal phenotypic plasticity and genotypic diversity affecting body growth and muscle cellularity in Atlantic salmon. Epigenetic variation in skeletal muscle growth is for the first time documented by presenting differences in DNA methylation and expression of *myogenin* in farmed Atlantic salmon families with either high or low breeding values for on-growth. The persistent effects of embryonic temperature on body growth and muscle cellularity were consistently shown to differ between two genotypes. Thermal plasticity and epigenetic variation in body growth are probably prerequisites for the local adaptation of salmon populations to fluctuating environmental conditions, and may potentially make them more resilient to global warming.

## Supporting information

S1 Fig *Myogenin* promoter sequence. Fourteen potential DNA methylation sites (bold CG) were identified in the proximal promoter region of Atlantic salmon *myogenin*. Five CG sites (green coloured) were assessed by pyrosequencing. Underlined sites indicate two E-boxes (CAnnTG) potentially binding myogenic regulatory factors. The promoter sequence ends with the ATG translational start site.
(TIF)

S1 Table Sampling dates and day-degrees for the developmental stages examined. Overview of date and day-degree (d°) for tissue sampling and weight registrations for the two temperature groups of 4°C and 8°C.
(DOCX)

S2 Table qPCR primers and pyrosequencing assays. Overview of primers used for qPCR and assays used for DNA methylation analysis by pyrosequencing. Pyrosequencing assays 1 and 5 provided a singular PCR product.
(DOCX)

S3 Table Results of GLM analysis. Main effects of type of on-growth (fast, slow) and incubation temperature (4°C, 8°C) and the interaction between genotype and incubation temperature on body weight measured at the five developmental stages, and for TGC and SHW.
(DOCX)

S4 Table Correlation analysis. Spearman correlation analysis between gene expression of *myogenin*, *dnmt1*, *dnmt3a* and *dnmt3b*, and the methylation levels of the five studied methylation sites (CpG). CpG1-5 refer to the putative CpG sites in the *myogenin* promoter located at -610, -598, -258, -255 and -234, respectively. *P*<0.05 are considered significantly different.
(DOCX)
