# Supplementary figures and images for: Genetic background and embryonic temperature affect DNA methylation and expression of myogenin and muscle development in Atlantic salmon (Salmo salar)
Source: PLoS One. 2017 Jun 29;12(6):e0179918. doi: 10.1371/journal.pone.0179918 (PMC5491062; doi:10.1371/journal.pone.0179918)

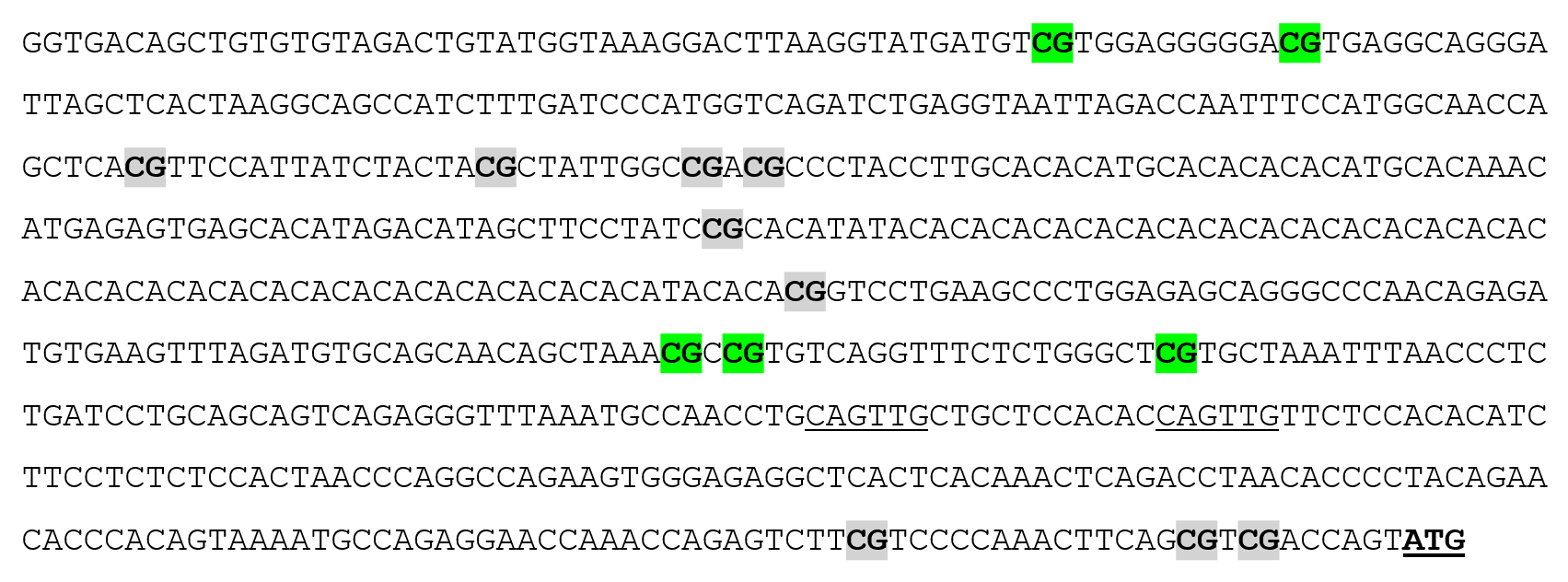

Supplement: S1 Fig — Fourteen potential DNA methylation sites (bold CG) were identified in the proximal promoter region of Atlantic salmon myogenin. Five CG sites (green coloured) were assessed by pyrosequencing. Underlined sites indicate two E-boxes (CAnnTG) potentially binding myogenic regulatory factors. The promoter sequence ends with the ATG translational start site. (TIF) [file pone.0179918.s001.tif]
