# Supplementary material for: Genetic background and embryonic temperature affect DNA methylation and expression of myogenin and muscle development in Atlantic salmon (Salmo salar)
Source: PLoS One. 2017 Jun 29;12(6):e0179918. doi: 10.1371/journal.pone.0179918 (PMC5491062; doi:10.1371/journal.pone.0179918)
Supplement: S1 Table — Overview of date and day-degree (d°) for tissue sampling and weight registrations for the two temperature groups of 4°C and 8°C. (DOCX) [file pone.0179918.s002.docx]

| **Stage** | **Date** | | **d° since fertilization** | | **Δ d°** |
| --- | --- | --- | --- | --- | --- |
|  | 4°C | 8°C | 4°C | 8°C |  |
| Eye pigmentation | 09.01.2014 | 23.11.2013 | 364 | 360 | 4 |
| Start feeding | 14.03.2014 | 30.01.2014 | 859 | 880 | -21 |
| Parr | 18.06.2014 | 23.04.2014 | 2085 | 1985 | 100 |
| Pre-smolt | 23.02.2015 | 21.10.2014 | 4164 | 3919 | 245 |
| Smolt | 16.03.2015 | 18.03.2015 | 4313 | 4597 | -284 |
| Sea-transfer | 22.04.2014 | 22.04.2014 | 4559 | 4687 | -128 |
| Harvest | 07.04.2016 | 07.04.2016 | 7947 | 8075 | -128 |
