# Supplementary material for: Genetic background and embryonic temperature affect DNA methylation and expression of myogenin and muscle development in Atlantic salmon (Salmo salar)
Source: PLoS One. 2017 Jun 29;12(6):e0179918. doi: 10.1371/journal.pone.0179918 (PMC5491062; doi:10.1371/journal.pone.0179918)
Supplement: S2 Table — Overview of primers used for qPCR and assays used for DNA methylation analysis by pyrosequencing. Pyrosequencing assays 1 and 5 provided a singular PCR product. (DOCX) [file pone.0179918.s003.docx]

| **qPCR primers** | |  |  |
| --- | --- | --- | --- |
| Gene |  | Sequence (5' → 3') | Efficiency* |
| *myogenin* | Forward | GTGCAAACGCAAGACTGTGAC | 2,00 |
|  | Reverse | TCCTCTTCAGGGCCTCGAAT |  |
| *dnmt1* | Forward | ACTGACTCTGCGCTGTCTTGTC | 2,01 |
|  | Reverse | CGTACTGCCCAGCCTGAAGT |  |
| *dnmt3a* | Forward | GAGGCTCGGCCCAAAGAG | 2,06 |
|  | Reverse | TGGCCACCACGTTTTCAA |  |
| *dnmt3b* | Forward | CGCAACATCACCAGAAAGAA | 2,00 |
|  | Reverse | AGCAGGCGGTAGAACTCAAA |  |
| **Pyrosequencing assay primers** | |  |  |
|  |  | Sequence (5' → 3') | Position CpG site |
| Assay 1 | Forward PCR Primer | GTGTAGATTGTATGGTAAAGGATTTAAG | -610, -598 |
|  | Reverse PCR Primer | CTACCTTAATAAACTAATCCCTACCTC |  |
|  | Sequencing Primer | AAGGATTTAAGGTATGATG |  |
| Assay 2 | Forward PCR Primer | GGTAGGGATTAGTTTATTAAGGTAGTT | -506; -489; -479; -476 |
|  | Reverse PCR Primer | CTTCACATCTCTATTAAACCCTACT |  |
|  | Sequencing Primer | ATTAATTTTTATGGTAATTAGT |  |
| Assay 3 | Forward PCR Primer | TGAGGTAGGGATTAGTTTATTAAGG | -404 |
|  | Reverse PCR Primer | CTTCACATCTCTATTAAACCCTACT |  |
|  | Sequencing Primer | ATGTATAAATATGAGAGTGAGT |  |
| Assay 4 | Forward PCR Primer | ATTTTATGGTTAGATTTGAGGTAATTAGAT | -326 |
|  | Reverse PCR Primer | CTTCACATCTCTATTAAACCCTACT |  |
|  | Sequencing Primer | CCTACTCTCCAAAACTT |  |
| Assay 5 | Forward PCR Primer | AGAGTAGGGTTTAATAGAGATGTGAA | -258; -255; -234 |
|  | Reverse PCR Primer | ATTTAAACCCTCTAACTACTACAAAATC |  |
|  | Sequencing Primer | AGTTTAGATGTGTAGTAATAGTTAA |  |
| Assay 6 | Forward PCR Primer | TAGAGGGTTTAAATGTTAATTTGTAGTT | -27; -11, -8 |
|  | Reverse PCR Primer | CTCTTAATAACCCCCCTCTAATC |  |
|  | Sequencing Primer | GTTAGAGGAATTAAATTAGAGTT |  |

*Efficiency=10^(-1/slope).
