# Supplementary material for: Genetic background and embryonic temperature affect DNA methylation and expression of myogenin and muscle development in Atlantic salmon (Salmo salar)
Source: PLoS One. 2017 Jun 29;12(6):e0179918. doi: 10.1371/journal.pone.0179918 (PMC5491062; doi:10.1371/journal.pone.0179918)
Supplement: S3 Table — Main effects of type of on-growth (fast, slow) and incubation temperature (4°C, 8°C) and the interaction between genotype and incubation temperature on body weight measured at the five developmental stages, and for TGC and SHW. (DOCX) [file pone.0179918.s004.docx]

**S3 Table. Results of GLM analysis.**

| **Dependent variable** | **Model** | **DF** | **Sum of Squares** | **Mean Square** | **F Value** | **Pr > F** |
| --- | --- | --- | --- | --- | --- | --- |
| **Start-feed** | Temp*On-growth | 3 | 0.0393 | 0.0131 | 7.52 | <.0001 |
|  | Temp | 1 | 0.0391 | 0.0391 | 22.52 | <.0001 |
|  | On-growth | 1 | 0.0001 | 0.0001 | 0.05 | 0.8204 |
|  |  |  |  |  |  |  |
| **Parr** | Temp*On-growth | 3 | 99.7243 | 33.2414 | 16.7 | <.0001 |
|  | Temp | 1 | 83.1417 | 83.1417 | 41.87 | <.0001 |
|  | On-growth | 1 | 16.1789 | 16.1789 | 8.15 | 0.0046 |
|  |  |  |  |  |  |  |
| **Pre-smolt** | Temp*On-growth | 3 | 98434.2518 | 32811.4173 | 12.22 | <.0001 |
|  | Temp | 1 | 47091.7909 | 47091.7909 | 17.24 | <.0001 |
|  | On-growth | 1 | 36098.2389 | 36098.2389 | 13.22 | 0.0003 |
|  |  |  |  |  |  |  |
| **Smolt** | Temp*On-growth | 3 | 51145220.2800 | 17048406.7600 | 2923.53 | <.0001 |
|  | Temp | 1 | 50401393.4600 | 50401393.4600 | 8645.06 | <.0001 |
|  | On-growth | 1 | 743701.7600 | 743701.7600 | 127.56 | <.0001 |
|  |  |  |  |  |  |  |
| **TGC FW** | Temp*On-growth | 3 | 0.8389 | 0.2796 | 95.12 | <.0001 |
|  | Temp | 1 | 0.8242 | 0.8242 | 292.51 | <.0001 |
|  | On-growth | 1 | 0.0147 | 0.0147 | 5.23 | 0.0313 |
|  |  |  |  |  |  |  |
| **Harvest** | Temp*On-growth | 3 | 987910041.5000 | 329303347.2000 | 289.24 | <.0001 |
|  | Temp | 1 | 161318426.8000 | 161318426.8000 | 141.29 | <.0001 |
|  | On-growth | 1 | 815258698.6000 | 815258698.6000 | 714.03 | <.0001 |
|  |  |  |  |  |  |  |
| **TGC SW** | Temp*On-growth | 3 | 163.5642 | 54.5214 | 372.73 | <.0001 |
|  | Temp | 1 | 84.2280 | 84.2280 | 572.4 | <.0001 |
|  | On-growth | 1 | 76.4565 | 76.4565 | 519.59 | <.0001 |
|  |  |  |  |  |  |  |
| **SHW** | Temp*On-growth | 3 | 964310607.8000 | 321436869.3000 | 392.75 | <.0001 |
|  | Temp | 1 | 477876480.6000 | 477876480.6000 | 582.89 | <.0001 |
|  | On-growth | 1 | 481199649.9000 | 481199649.9000 | 586.95 | <.0001 |
|  |  |  |  |  |  |  |
| **ΔSHW** | On-growth | 1 | 95656.2508 | 95656.2508 | 3.35 | 0.09 |
