# Supplementary material for: Genetic background and embryonic temperature affect DNA methylation and expression of myogenin and muscle development in Atlantic salmon (Salmo salar)
Source: PLoS One. 2017 Jun 29;12(6):e0179918. doi: 10.1371/journal.pone.0179918 (PMC5491062; doi:10.1371/journal.pone.0179918)
Supplement: S4 Table — Spearman correlation analysis between gene expression of myogenin, dnmt1, dnmt3a and dnmt3b, and the methylation levels of the five studied methylation sites (CpG). CpG1-5 refer to the putative CpG sites in the myogenin promoter located at -610, -598, -258, -255 and -234, respectively. P<0.05 are considered significantly different. (DOCX) [file pone.0179918.s005.docx]

| **S4 Table. Correlation analysis.** | | | | | | | | | | | |
| --- | --- | --- | --- | --- | --- | --- | --- | --- | --- | --- | --- |
|  | | | *myogenin* | *dnmt1* | *dnmt3a* | *dnmt3b* | CpG1 | CpG2 | CpG3 | CpG4 | CpG5 |
|  | *myogenin* | ρ | 1.000 | .839^**^ | .762^**^ | .770^**^ | -.616^**^ | -.615^**^ | -.583^**^ | -.409^**^ | -.412^**^ |
|  |  | p-value | . | .000 | .000 | .000 | .000 | .000 | .000 | .000 | .000 |
|  |  | N | 143 | 140 | 138 | 134 | 130 | 130 | 125 | 125 | 123 |
|  | *dnmt1* | ρ | .839^**^ | 1.000 | .629^**^ | .755^**^ | -.607^**^ | -.615^**^ | -.640^**^ | -.442^**^ | -.412^**^ |
|  |  | p-value | .000 | . | .000 | .000 | .000 | .000 | .000 | .000 | .000 |
|  |  | N | 140 | 140 | 136 | 132 | 127 | 127 | 122 | 122 | 120 |
|  | *dnmt3a* | ρ | .762^**^ | .629^**^ | 1.000 | .589^**^ | -.404^**^ | -.383^**^ | -.369^**^ | -.219^*^ | -.333^**^ |
|  |  | p-value | .000 | .000 | . | .000 | .000 | .000 | .000 | .016 | .000 |
|  |  | N | 138 | 136 | 138 | 132 | 125 | 125 | 120 | 120 | 118 |
|  | *dnmt3b* | ρ | .770^**^ | .755^**^ | .589^**^ | 1.000 | -.472^**^ | -.462^**^ | -.475^**^ | -.304^**^ | -.301^**^ |
|  |  | p-value | .000 | .000 | .000 | . | .000 | .000 | .000 | .001 | .001 |
|  |  | N | 134 | 132 | 132 | 134 | 125 | 125 | 117 | 117 | 115 |
|  | CpG1 | ρ | -.616^**^ | -.607^**^ | -.404^**^ | -.472^**^ | 1.000 | .973^**^ | .754^**^ | .687^**^ | .566^**^ |
|  |  | p-value | .000 | .000 | .000 | .000 | . | .000 | .000 | .000 | .000 |
|  |  | N | 130 | 127 | 125 | 125 | 155 | 155 | 133 | 133 | 130 |
|  | CpG2 | ρ | -.615^**^ | -.615^**^ | -.383^**^ | -.462^**^ | .973^**^ | 1.000 | .760^**^ | .701^**^ | .569^**^ |
|  |  | p-value | .000 | .000 | .000 | .000 | .000 | . | .000 | .000 | .000 |
|  |  | N | 130 | 127 | 125 | 125 | 155 | 155 | 133 | 133 | 130 |
|  | CpG3 | ρ | -.583^**^ | -.640^**^ | -.369^**^ | -.475^**^ | .754^**^ | .760^**^ | 1.000 | .874^**^ | .829^**^ |
|  |  | p-value | .000 | .000 | .000 | .000 | .000 | .000 | . | .000 | .000 |
|  |  | N | 125 | 122 | 120 | 117 | 133 | 133 | 161 | 160 | 157 |
|  | CpG4 | ρ | -.409^**^ | -.442^**^ | -.219^*^ | -.304^**^ | .687^**^ | .701^**^ | .874^**^ | 1.000 | .793^**^ |
|  |  | p-value | .000 | .000 | .016 | .001 | .000 | .000 | .000 | . | .000 |
|  |  | N | 125 | 122 | 120 | 117 | 133 | 133 | 160 | 160 | 157 |
|  | CpG5 | ρ | -.412^**^ | -.412^**^ | -.333^**^ | -.301^**^ | .566^**^ | .569^**^ | .829^**^ | .793^**^ | 1.000 |
|  |  | p-value | .000 | .000 | .000 | .001 | .000 | .000 | .000 | .000 | . |
|  |  | N | 123 | 120 | 118 | 115 | 130 | 130 | 157 | 157 | 157 |
| **. Correlation is significant at the 0.01 level (2-tailed). | | | | | | | | | | | |
| *. Correlation is significant at the 0.05 level (2-tailed). | | | | | | | | | | | |
